# Multi-particle quantum walks on 3D integrated photonic chip

**DOI:** 10.1038/s41377-024-01627-7

**Published:** 2024-10-19

**Authors:** Wen-Hao Zhou, Xiao-Wei Wang, Ruo-Jing Ren, Yu-Xuan Fu, Yi-Jun Chang, Xiao-Yun Xu, Hao Tang, Xian-Min Jin

**Affiliations:** 1 Center for Integrated Quantum Information Technologies (IQIT), School of Physics and Astronomy and State Key Laboratory of Advanced Optical Communication Systems and Networks, Shanghai Jiao Tong University, Shanghai, 200240 China; 2 Hefei National Laboratory, Hefei, 230088 China; 3 School of Artificial Intelligence Science and Technology, University of Shanghai for Science and Technology, Shanghai, China; 4 Institute of Photonic Chips, University of Shanghai for Science and Technology, Shanghai, China; 5 Chip Hub for Integrated Photonics Xplore (CHIPX), Shanghai Jiao Tong University, Wuxi, 214000 China; 6 TuringQ Co., Ltd., Shanghai, 200240 China

**Keywords:** Single photons and quantum effects, Quantum optics

## Abstract

Quantum walks provide a speed-up in computational power for various quantum algorithms and serve as inspiration for the construction of complex graph representations. Many pioneering works have been dedicated to expanding the experimental state space and the complexity of graphs. However, these experiments are mostly limited to small experimental scale, which do not reach a many-body level and fail to reflect the multi-particle quantum interference effects among non-adjacent modes. Here, we present a quantum walk with three photons on a two-dimensional triangular lattice, which is mapped to a 19 × 19 × 19 high-dimensional state space and constructs a complex graph with 6859 nodes and 45,486 edges. By utilizing the statistical signatures of the output combinations and incorporating machine learning techniques, we successfully validate the nonclassical properties of the experiment. Our implementation provides a paradigm for exponentially expanding the state space and graph complexity of quantum walks, paving the way for surmounting the classical regime in large-scale quantum simulations.

## Abstract (web summary)

Constructing complex graph representations through multi-photon quantum walks on the integrated photonic platform

**Graphical Abstract:**

## Graphical abstract

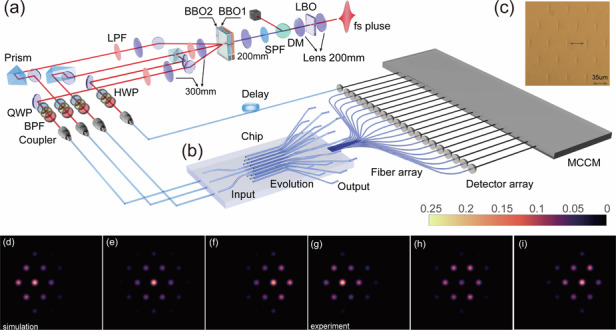

## Introduction

Classical random walks and Markov chains on graphs have been used as computational frameworks for developing algorithms in mathematics, physics, computer science, and economics^1^. In contrast to classical random walks, quantum walks (QWs)^2,3^, based on inherent quantum superposition, can be applied to realize quantum algorithms^4–8^ and facilitate the development of quantum simulation^9–11^, quantum transport processes^12–14^, quantum random number^15,16^ and universal quantum computation^17,18^. Quantum walks have been demonstrated on various physical platforms, including trapped neutral atoms^19^, trapped ions^20–22^, nuclear magnetic resonance^23^, superconducting systems^24,25^, and bulk and fiber optics^26,27^. The emerging integrated photonic platform^28–36^ is regarded as a promising candidate for realizing high-dimensional quantum walks, benefiting from the robust and long-coherent nature of photons.

Quantum walks provide a framework for studying the transport properties of complex connected graphs, whose complexity depends on the node number and the edge number. Complex graphs with a high degree of connectivity offer potential applications^37^, including the graph isomorphism problems^38^, quantum search algorithms^39,40^ and multi-partite quantum communication^41^. The complexity of the graph can be expanded by increasing the experimental physical and synthetic dimensions, such as spatial mode, momentum, polarization, and temporal loop^42–49^, and the graph structure can also be programmed through the quantum walk processor^50^. Another direct way is to increase the number of quantum walkers, which can exponentially increase the state space and graph complexity. However, most experiments are limited to two correlated photons and a few modes, primarily constrained by the detection and validation of multi-particle quantum interference. Particularly in high-dimensional quantum walks, it is difficult to sample all the combinations, including collision-free events and quantum bunching events. Therefore, efficient and reliable methods to witness the nonclassical correlations arising from multi-particle quantum interference are necessary.

In this work, we experimentally demonstrate a quantum walk of three photons on a 3D (2D lattice and 1D time evolution) photonic chip, which can be mapped to a complex graph with an extraordinarily high degree of connectivity and complexity. The triangular lattice is connected to the 2D fanout fiber array through site-by-site coupling technology^29^. We inject three indistinguishable photons into the chip and observe obvious nonclassical correlations and multi-photon quantum interferences among non-adjacent modes. We collect complete collision-free combinations and part of quantum bunching combinations within a moderate experimental execution time. By using the statistical signatures of the output combinations and machine learning techniques, we successfully verify the difference between quantum and classical behavior in three-photon quantum walks.

## Results

### Graph represents of the three-photon quantum walks

**Fig. 1:**
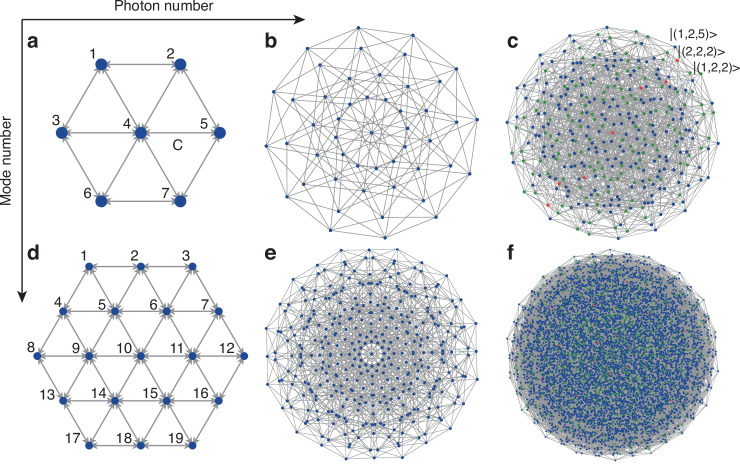
Schematic of graph representations in different dimensions. **a** Single-layer triangular lattice. The central site is connected to six surrounding sites, and all connections have the same coupling strength *C*. **b**, **c** Sketch of high-dimensional graph representations of a single-layer lattice spanned by 2-photon and 3-photon injection. Each node represents a multi-particle state, and every edge represents a possible transition between multi-particle states. All the nodes are divided into three groups: collision-free events (blue), two-photon bunching events (green) and three-photon bunching event (red). **d** Two-layer triangular lattice. The experimental structure consists of 19 sites. **e**, **f** Expanded graph representations of a two-layer lattice spanned by 2-photon and 3-photon injection, corresponding to 361 nodes, 1596 edges and 6859 nodes, 45,486 edges, respectively

Compared with other lattices, the triangular lattice has higher node connectivity^51^ and results in higher graph complexity. Figure 1a shows a single-layer triangular lattice, where the central site has six edges coupling to its neighbors, and all the coupling strengths are *C*. By calculating the Cartesian product of Fig. 1a with itself, we can obtain the high-dimensional graphs corresponding to 2-photon and 3-photon state injection.

As shown in Fig. 1, increasing the photon number offers a scalable approach to enhance graph complexity and connectivity, which can be observed through the node and edge number on the graphs. When comparing the 2-photon and the 3-photon quantum walks on a single layer triangular lattice in Fig. 1b, c respectively, the constructed graphs consist of 49 nodes, 168 edges and 343 nodes, 1764 edges, respectively. Each node in the graph represents a multi-photon basis state. For the 3-photon case, we further categorize the nodes in Fig. 1c into three groups: blue nodes represent the collision-free events (e.g., $\left\vert (1,2,5)\right\rangle$); green nodes represent two-photon bunching events (e.g., $\left\vert (1,2,2)\right\rangle$); and red nodes represent three-photon bunching events. (e.g., $\left\vert (2,2,2)\right\rangle$), where (*i*, *j*, *k*) represents an output combination. The gray edges represent the transitions and hops between two states which differ in only one nearest site, such as $\left\vert (1,2,2)\right\rangle$ and $\left\vert (1,2,5)\right\rangle$, or $\left\vert (1,2,2)\right\rangle$ and $\left\vert (2,2,2)\right\rangle$.

In addition to increasing the photon number, increasing mode number can also expend the graph complexity. We extend the lattice to two layers, and the structure has been shown in Fig. 1d. Similarly, Fig. 1e, f show the high-dimensional graphs spanned by the multi-photon state injection. The graph constructed in our experiment is shown in Fig. 1f, which includes 6859 nodes and 45,486 edges. We also categorize the nodes into three groups using different colors to distinguish different states. Considering the photon permutation symmetry, the 6859 nodes can be decomposed into 6859 = 969 × 6 + 342 × 3 + 19, comprising 969 collision-free combinations, 342 two-photon bunching combinations and 19 three-photon bunching combinations in our experiment. The enlarged state space $\left\vert \psi \right\rangle$ and graph connectivity grow when the photon number and mode number increase.

### Experimental demonstration of the three-photon quantum walks

**Fig. 2:**
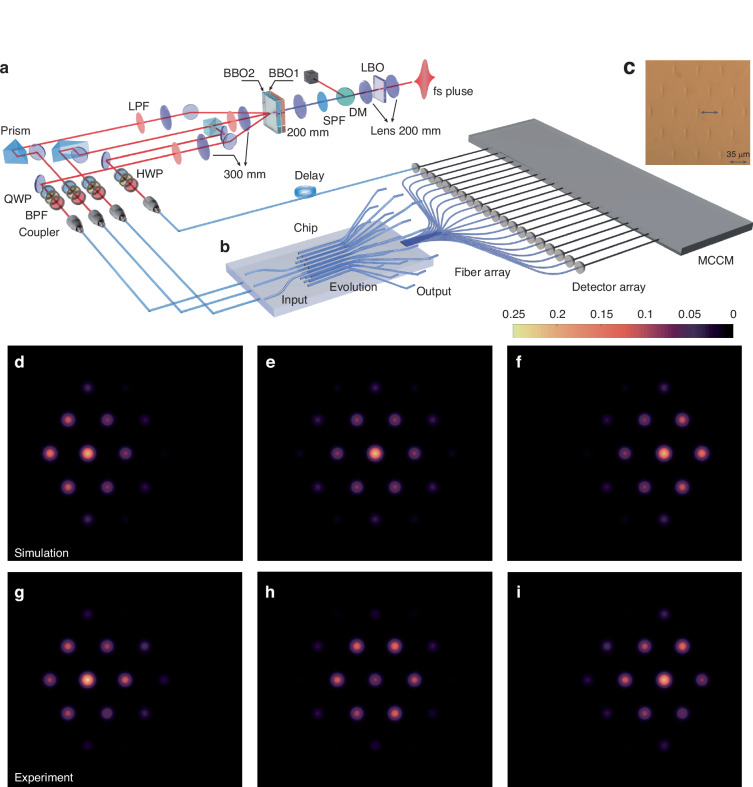
Experimental setup and photon intensity distributions. **a** Experimental setup diagram. The experimental setup includes quantum state preparation, on-chip unitary operation, and large-scale correlation measurement. **b** Waveguide structure. The 3D evolution region (2D lattice and 1D time evolution) smoothly transitions to the outputs and then connects to an external fiber array using site-by-site coupling technology. **c** The fabricated waveguide cross-section at the output of the chip. **d**–**f** Simulation results of the single-photon intensity distribution. **g**–**i** Experimental results of the single-photon intensity distribution by avalanche photodetectors

The experimental setup is shown schematically in Fig. 2a, including quantum state preparation, on-chip unitary operation, and large-scale correlation measurement. A femtosecond pulse (140 fs) with a 780 nm central wavelength (Chameleon Vision, Coherent) is frequency-doubled by a lithium triborate (LBO) crystal. The dichroic mirror (DM) and short pass filter are used to filter out the residual 780 nm laser. The generated 390 nm laser pulse is then used to pump two beta barium borate (BBO) crystals, generating two pairs of correlated photons via spontaneous parametric down-conversion^52–54^. The BBO1 is type-II collinear phase-matched configuration and BBO2 is type-II phase matching in a beam-like scheme. The polarizations of the generated photons are initialized (horizontal polarization) using quarter wave plates and half wave plates. The 3 nm band pass filters are employed to ensure the near-identical spectrums. Then, three photons are coupled into polarization-maintaining (PM) optical fibers and injected into the photonic chip via a PM V-groove fiber array, while the remaining one serves as the trigger signal. The temporal indistinguishability of non-classical interference among different down-converted photons is achieved by translating prisms on the linear motorized stages to compensate external delays (see Supplementary Material S1 for interference results). The outputs of the waveguides are connected to the 2D multi-mode V-groove fiber array and subsequently connected to the avalanche photodetectors. All the electronic signals are sent to a self-developed multi-channel coincidence module, which can record all the output combination events simultaneously.

The triangular lattice in the photonic chip is realized using the femtosecond laser direct-writing technique^55^ (see Methods for fabrication details). The waveguide structure inside the chip is shown in Fig. 2b, where three input waveguides are coupled into the chip and then expand to the outputs after evolution. The waveguide spacing in the coupling region is 15 μm, and the number of sites is *N* = 19. The spacings of the input and output waveguides are set to 127 μm and 35 μm respectively, to match the external fiber array. The fabricated waveguide cross-section at the output of the chip is shown in Fig. 2c and the waveguide diameter size can be estimated from the legend in Fig. 2c. Compared to the 1D waveguide array, the advantages of 3D fabrication allow for efficient expansion of the structure size and experimental scale, providing an excellent platform for multi-photon experiments such as quantum walks and boson sampling^56–58^.

We characterize the chip by injecting heralded single photons into the three inputs and then we reconstruct the intensity distribution of photons after the evolution of 2 mm (see Supplementary Material S2 for evolution length details). Fig. 2d–f show the simulation results, while Fig. 2g–i display the experimental results (see Methods for simulation details). We calculate the similarity ${S}_{i}={\left({\sum }_{j}\sqrt{{p}_{i,j}^{exp}{p}_{i,j}^{th}}\right)}^{2}/\left({\sum }_{j}{p}_{i,j}^{exp}{\sum }_{j}{p}_{i,j}^{th}\right)$ between the two intensity distribution ${p}_{i,j}^{exp}$ and ${p}_{i,j}^{th}$, where *p*_*i*,*j*_ is the probability of detecting one photon at output *j* when we excite input *i*. We calculate the similarity and corresponding uncertainty results, which are 97.50% ± 0.01%, 93.74% ± 0.02%, and 97.00% ± 0.02% (see Supplementary Material S2 for deviation analysis). The uncertainties are computed from single-photon intensity detection, assuming Poissonian statistics. In the experiment, we reduce the experimental uncertainties by accumulating counts.

### Correlation measurements of the three-photon quantum walks

Three-photon quantum walks can be described by the unitary evolution operator *U* = *e*^−*iHz*^, where *z* is the evolution length and *H* is the Hamiltonian determined by waveguide structure. The initial state of the experiment is encoded in the three-photon Fock state $\left\vert 1,1,1\right\rangle$. For the case of three outputs, the corresponding output state after the evolution induced by *U* can be expressed as: $\left|1,1,1\right\rangle \mathop{\rightarrow}\limits^{U} P_{(1,1,1)}\left|1,1,1\right\rangle+P_{(2,1,0)}\left|2,1,0\right\rangle+P_{(3,0,0)}\left|3,0,0\right\rangle$^59^. The different output Fock states $\left\vert 1,1,1\right\rangle$, $\left\vert 2,1,0\right\rangle$ and $\left\vert 3,0,0\right\rangle$ correspond to collision-free events, two-photon bunching events and three-photon bunching events, respectively. In the simulation, we can derive a 3 × 3 submatrix *M* from the matrix *U* for a specific output combination and the probability *P*_(*i*, *j*, *k*)_ of an event is linked to the permanent of the submatrix *M*.

In the experiment, for the multi-photon classical correlation, we can simply perform an incoherent summation of the intensity distribution extracted from Fig. 2d–f to estimate, which cannot demonstrate any quantum features^60^. To describe the multi-photon interferences in the enlarged state space $\left\vert \psi \right\rangle$, the three-photon quantum correlation among outputs (*i*, *j*, *k*) can be defined as:

1 $${\Gamma }_{i,j,k}=\left\langle \psi \right\vert {a}_{i}^{{\dagger} }{a}_{j}^{{\dagger} }{a}_{k}^{{\dagger} }{a}_{k}{a}_{j}{a}_{i}\left\vert \psi \right\rangle$$

where ${a}_{i}^{{\dagger} }({a}_{i})$ is the bosonic creation (annihilation) operator for a photon at output *i*.

**Fig. 3:**
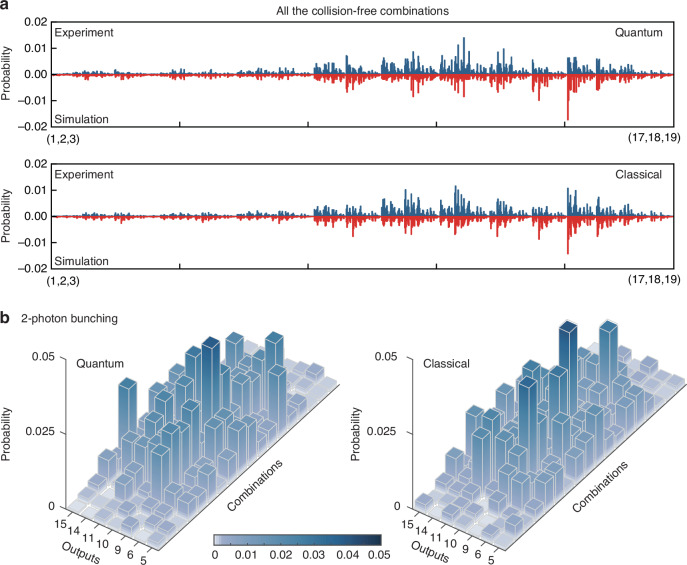
Simulated and measured three-photon correlations. **a** Experimental and theoretical correlations of indistinguishable and distinguishable photons with all collision-free combinations. The horizontal axes represent all the output combinations from (1,2,3) to (17,18,19). The fidelities *F* are 93.42% ± 0.15% and 95.70% ± 0.14%. **b** Experimental correlations of indistinguishable and distinguishable photons in the subspace of the 2-photon bunching events (7 groups: site 5,6,9,10,11,14,15). Each group includes 18 bunching combinations and all the counts are accumulated with 15 h to obtain the normalized probability distributions. Some parts show an increase in quantum correlations compared to classical correlations induced by multi-photon interferences

We collect the complete collision-free combinations with 15 h and normalize the counts to calculate the probability distribution. The quantum and classical correlations are experimentally and theoretically depicted in Fig. 3a. The fidelity $F={\sum }_{i,j,k}\sqrt{{\Gamma }_{i,j,k}^{exp}{\Gamma }_{i,j,k}^{sim}}$ can be used to characterize the deviation between simulation and experiment, resulting in calculated values of 93.42% ± 0.15% and 95.70% ± 0.14% for indistinguishable and distinguishable photons, respectively. The deviation mainly arises from the differential losses of varying waveguide bending radii during the bending transformations, which can be improved by employing imaging-based large-scale correlation measurement.

Multi-photon interference has been observed in different structures with a few modes^59,61–63^. In large-scale quantum walks, multi-photon interference establishes nonclassical correlations among non-adjacent sites, leading to quantum bunching effects. Therefore, it is important to collect 2-photon and 3-photon bunching events among different modes. However, the proportion of bunching events decreases rapidly as mode number increases. In our experiment, the proportions of 2-photon and 3-photon bunching combinations are 25.7% and 1.4%, respectively (see Supplementary Material S3 for calculation details). As a result, we focus on collecting the most representative bunching events within the moderate experimental runtime.

**Fig. 4:**
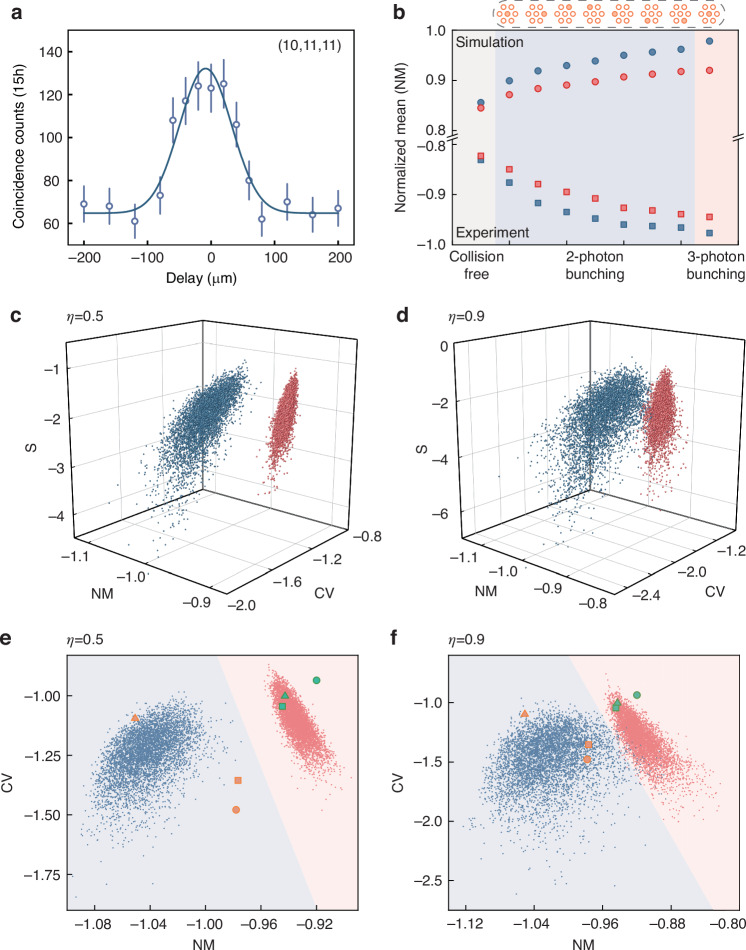
Nonclassical certification of three-photon quantum walks. **a** Two-photon bunching curve of the combination (10,11,11). **b** Simulation and experimental statistical signatures NM of the *C*-dataset with indistinguishable *I* (blue) and distinguishable *D* (red) photons. The graph within the dashed box represents the corresponding added bunching events, which create a separation between the quantum and classical results. **c**, **d** Validation results in the (NM, CV, S) space with different *η* by numerically simulating 5000 different Hamiltonians. *η* represents the degree of perturbation. (**e**, **f**) Validation results in the (NM, CV) plane with different *η*. According to the SVM classification algorithm, the experimental (square) and simulation (circle) points can fall into one of two clouds representing *I* (blue) and *D* (red) photons. The triangle points represent the theoretical values without perturbation, based on the complete correlation matrix

For the 2-photon bunching events, we introduce a balanced beamsplitter at each output one by one and measure the quantum and classical correlations of each group for a duration of 15 hours (see Supplementary Material S3 for measurement details). We only detect a sufficient number of coincidence counts at the first layer of the triangular lattice (site 5, 6, 9, 10, 11, 14, 15). We then reconstruct the counts and normalize the probability distribution in the subspace. The results are presented in Fig. 3b, where we observe that some of the quantum correlations increase due to the bunching effect. In Fig. 4a, we show a complete interference curve of the bunching combination (10,11,11) by shifting the delay interval, and the peak value is nearly twice that of the distinguishable case. For the 3-photon bunching events, we add a cascade of fiber beamsplitters to separate the photons. After a collection time of 50 h, we obtain 23 counts for classical correlation and 65 counts for quantum correlation only at central site 10 and we cannot collect meaningful data at other sites due to their extremely low occurrence probability. As the scale of the experiment increases, the complete measurement of all the bunching combinations is challenging due to low count rate and occurrence probability, so probability distributions alone may not offer a definitive distinction between classical and quantum walks. Therefore, it is important to validate the quantum features of the experiment with limited bunching events.

### Validation of the quantum features in three-photon quantum walks

To obtain a comprehensive understanding of the role of multi-photon interference, several validation protocols have been proposed to verify the nonclassical nature of quantum correlation, but their applicability is limited in certain scenarios. For example, we use the likelihood ratio test^64^ to distinguish boson samplers from distinguishable samplers, but in practice, the validation can still pass even without the occurrence of bunching events. Additionally, variance and Pólya number^28^ are often used to quantify the transport properties of single-photon quantum walks and the Cauchy-Schwarz inequality^65^ is often employed to test 2-photon quantum correlations, but these validations have no higher-order correspondences.

It has been proven that the quantum characteristics of multi-photon interference can be demonstrated through lower-order correlations^66^, and the validation process is based on the evaluation of statistical signatures from the *C*-dataset. The lower-order correlation function *C*_*mn*_ can be defined as follows:

2 $${C}_{mn}=\left\langle {\hat{n}}_{m}{\hat{n}}_{n}\right\rangle -\left\langle {\hat{n}}_{m}\,\right\rangle \left\langle {\hat{n}}_{n}\right\rangle$$

where $\left\langle {\hat{n}}_{m}{\hat{n}}_{n}\right\rangle (\left\langle {\hat{n}}_{m}\,\right\rangle )$ represents the coincidence (single) detection events.

To estimate the 2-mode correlation statistics from the 3-photon experiment, we introduce the quantity ${{{\mathcal{N}}}}_{ijk}$ which represents the 3-photon coincidence counts for a specific output (*i*, *j*, *k*). Then, we evaluate the 2-mode correlation using the following equation^67^:

3 $$\begin{array}{l}\left\langle {\hat{n}}_{m}{\hat{n}}_{n}\right\rangle \simeq \frac{1}{N}\mathop{\sum}\limits_{k\ge j\ge i}{n}_{ijk}^{m}{n}_{ijk}^{n}{{{\mathcal{N}}}}_{ijk}\\ \left\langle {\hat{n}}_{m}\right\rangle \simeq \frac{1}{N}\mathop{\sum}\limits_{k\ge j\ge i}{n}_{ijk}^{m}{{{\mathcal{N}}}}_{ijk}\end{array}$$

Here, *N* is the total sample size, which is about 1 × 10^5^ with 15 h. ${n}_{ijk}^{m}$ is the eigenvalue of the number operator in mode *m* of the output (*ijk*). In detail, ${n}_{ijk}^{m}=1$ for collision-free events, ${n}_{ijk}^{m}=2$ for 2-photon bunching events, and ${n}_{ijk}^{m}=3$ for 3-photon bunching events. After completing these preparations, we obtain the *C*-dataset.

In Fig. 4b, we perform statistical analysis on the dataset with normalized mean NM (the expected value divided by *n*/*m*^2^) and present the simulated values above the axes for visualization purposes. In Fig. 4b, solely utilizing the ${{{\mathcal{N}}}}_{ijk}$ containing collision-free combinations to calculate NM does not provide a clear differentiation between quantum (blue) and classical (red) walks. However, when we incrementally incorporate two-photon bunching events come from Fig. 3b and three-photon bunching events at central site 10, we observe a clear separation between the quantum and classical results, and the absolute value of quantum NM is always larger than the classical NM. The separation of NM emphasizes the significance of bunching events in distinguishing quantum and classical correlations, particularly when only partial bunching events (two-photon bunching events at the first layer of the triangular lattice and three-photon bunching event at central site 10) is present. This protocol proves to be a valuable tool for quantifying the distinction between classical and quantum walks.

We extend the validation to the (NM, CV, S) space^68^, where CV is the standard deviation divided by the expectation of the distribution, and S is the skewness. By perturbing the positions of the sites, we simulate 5000 instances of quantum walks with different Hamiltonians and obtain 5000 sets of C-datasets. We define the perturbation degree *η* (see Supplementary Material S4 for details) and simulate two cases: moderate perturbation range (*η* = 0.5) and large perturbation range (*η* = 0.9). An increase in *η* indicates that the triangular lattice becomes more unstable. In Fig. 4c, d, with increasing *η*, the discrimination between quantum and classical regions becomes blurred, especially in terms of skewness. However, even in this scenario, the validation method effectively distinguishes between quantum (blue) and classical (red) regions, demonstrating its robustness.

Furthermore, we project the simulation results from the quantum (blue) and classical (red) clouds onto the (NM, CV) plane. We use the support vector machine (SVM) algorithm to distinguish between quantum and classical regions and simulate the perturbation-free theoretical values (triangle) using the complete correlation matrix. In Fig. 4e, f, we observe correct classification for both theoretical (circle) and experimental (square) results, using the partial correlations shown in Fig. 3. This validation method reflects the holistic quantum features of correlation measurements rather than focusing on specific values, rendering it resilient to the experimental fidelity deviations, providing strong evidence for testing quantum features of multi-photon quantum walks.

## Discussion

In summary, we experimentally conduct a quantum walk of three photons on a 3D photonic chip, which can be mapped to a complex graph with 6859 nodes and 45,486 edges. Two-dimensional lattice containing three identical photons exhibits a unique graph that transcends two dimensions. We choose the triangular lattice which has higher node connectivity to construct complex graphs, which play a central role in realizing quantum simulations and quantum computation, such as the graph isomorphism problem^38^ and universal quantum computation^41^. Importantly, we can adjust the structure of the two-dimensional waveguide array to correspond to different Hamiltonians and analog quantum computation problems, such as quantum spatial search^30^ and quantum fast hitting^69^. When we extend these quantum computing tasks to multi-photon experiments, the presence of multi-photon quantum correlations and bunching effect (see Supplementary Material S5 for details) potentially enhance the search efficiency or hitting probability on a single marked site.

Furthermore, by calculating the statistical signatures of the *C*-dataset, we observe the strong evidence that distinguishes between quantum and classical features in quantum walks. The statistical indicator NM provides a protocol for assessing genuine multi-photon interference in large-scale quantum systems. We perform statistical analysis on the datasets with NM, CV, and S, and visualize the outcomes in both three-dimensional and two-dimensional spaces, forming distinct quantum and classical clouds. We verify the results derived from the correlation matrix, encompassing complete collision-free events and partial bunching events correctly fall within the separation regions. This validation method requires only a subset of collision-free and bunching events, making it very suitable for experiments that aim to achieve quantum advantage or noisy intermediate-scale quantum (NISQ) technology^70^.

## Materials and methods

### 3D photonic chip fabrication

**Fig. 5:**
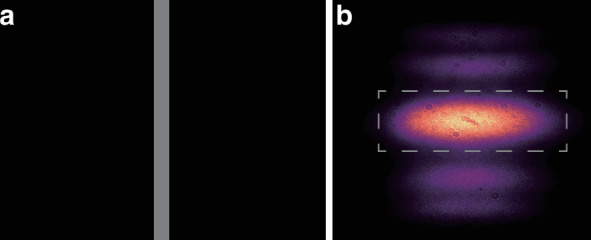
Phase diagram of the spatial light modulator. The spatial light modulator is used to induce diffraction, and then we use slit to select the 0-level diffraction fringe in order to make the written waveguide transmission mode more Gaussian

The 3D photonic chip is fabricated by femtosecond laser direct-writing technology. The femtosecond pulse (290 fs) with a 1026 nm central wavelength (Pharos-10W, Light Coversion) is frequency-doubled by the second harmonic module integrated in the laser, resulting in a laser wavelength of 513 nm. The generated femtosecond laser (1 MHz repetition frequency, 290 fs pulse duration) is then shaped by the spatial light modulator (single slit). The purpose of beam shaping is to make the written waveguide transmission mode more Gaussian. We use the spatial light modulator to induce diffraction, and then use slit to select the 0-level diffraction fringe, as shown in Fig. 5. Finally, The light spot is focused into a 5 cm borosilicate glass (Eagle XG, Corning) substrate with the 50 × objective lens (0.55 NA, M plan Apo, Mitutoyo). The chip is adsorbed on the high-precision air-bearing platform and the waveguide structure is fabricated by moving the platform. We scan different laser powers and platform translation velocities, and determine that optimal single-mode and Gaussian waveguide properties are achieved at 80 mW power and 10 mm s^−1^ velocity. Power compensation is employed to maintain the similar waveguide constant *β* at different chip depths. In addition, transforming the 2D array to the 1D output array introduces random coupling between different waveguides, while the 2D bending transformation in Fig. 2b can be seen as a gradual coupling process, making it more suitable for Hamiltonian engineering in quantum walks.

### Intensity distribution of continuous-time quantum walks

The Hamiltonian of the photons propagating through evanescently coupled waveguides can be defined as:

4 $$H=\mathop{\sum }\limits_{i=1}^{N}{\beta }_{i}{a}_{i}^{{\dagger} }{a}_{i}+\mathop{\sum }\limits_{i\ne j}^{N}{C}_{i,j}{a}_{i}^{{\dagger} }{a}_{j}$$

where *β*_*i*_ is the propagation constant of the site *i*, *C*_*i*,*j*_ is the coupling strength between site *i* and *j*, and ${a}_{i}^{{\dagger} }({a}_{i})$ is bosonic creation (annihilation) operator for site *i*. *i* and *j* range from 1 to *N*. In the experiment, we only consider the coupling that happens between two nearest sites. For instance, the central site 10 has six nearest linkages and all the coupling strength is set as *C*. In our experiment, the photonic chip contains two parts of the Hamiltonian, the time-independent Hamiltonian *H*_1_ in the evolution stage and the time-dependent Hamiltonian *H*(*t*)_2_ in the bending transformation stage. For the bending transformation stage, we use the differential element method to split it into small segments and assume that the Hamiltonian in each segment is time-independent. Then, the unitary evolution operator can be expressed as *U* = *exp*(−*iHz*), where *z* is the evolution length. By applying the unitary evolution operator on the input operator ${a}_{i}^{{\dagger} }$, we can calculate the results of the equation on the output operator ${a}_{j}^{{\dagger} }(z)=\mathop{\sum }_{i = 1}^{N}{U}_{i,j}(z){a}_{i}^{{\dagger} }(0)$ and the intensity distribution of single-photon in site *j* is calculated by $\left\vert {U}_{i,j}(z){U}_{i,j}^{{\dagger} }(z)\right\vert$.

## Supplementary information

Supplemental Materials for Multi-particle quantum walks on 3D integrated photonic chip

## Data Availability

The data that support the findings of this study are available from the corresponding authors on reasonable request.
