# Supplementary material for: Multi-particle quantum walks on 3D integrated photonic chip
Source: Light Sci Appl. 2024 Oct 19;13:296. doi: 10.1038/s41377-024-01627-7 (PMC11489590; doi:10.1038/s41377-024-01627-7)
Supplement: Supplementary file 1 — Supplemental Materials for Multi-particle quantum walks on 3D integrated photonic chip [file 41377_2024_1627_MOESM1_ESM.pdf]

Supplementary Information for: Multi-particle quantum walks on 3D integrated photonic chip

Wen-Hao Zhou,[1,2] Xiao-Wei Wang,[1,2] Ruo-Jing Ren,[1,2,5,6] Yu-Xuan Fu,[1,2] Yi-Jun Chang,[1,2] Xiao-Yun Xu,[1,2,4] Hao Tang,[1,2] Xian-Min Jin[1,2,3,4†]
[1]Center for Integrated Quantum Information Technologies (IQIT),
School of Physics and Astronomy and State Key Laboratory of Advanced
Optical Communication Systems and Networks,
Shanghai Jiao Tong University, Shanghai 200240, China.
[2]Hefei National Laboratory, Hefei 230088, China
[3]TuringQ Co., Ltd., Shanghai 200240, China
[4]Chip Hub for Integrated Photonics Xplore (CHIPX),
Shanghai Jiao Tong University, Wuxi 214000, China
[5]School of Artificial Intelligence Science and Technology,
University of Shanghai for Science and Technology, Shanghai, China
[6]Institute of Photonic Chips,
University of Shanghai for Science and Technology, Shanghai, China
[†]E-mail: xianmin.jin@sjtu.edu.cn

S.1 Preparation of 4-photon quantum source

We first prepare two pairs of correlated photons via spontaneous parametric down-conversion (SPDC). Then three photons are injected into the chip, and the remaining photon acts as the trigger. Since the two pairs of quantum sources are independent, the four-photon Fock state can be decomposed into the direct product of the two pairs of quantum sources: $|1,1,1,1\rangle = |1,1\rangle \otimes |1,1\rangle$. In the experiment, we obtain the four-photon Fock state by synchronizing two pairs of quantum sources.

We present the interference visibilities of individual pairs of correlated photons and the interference visibilities of synchronizing two pairs of quantum sources by scanning delay as illustrated in Fig.S1. When the laser pump power is 150 mW, the interference visibilities of

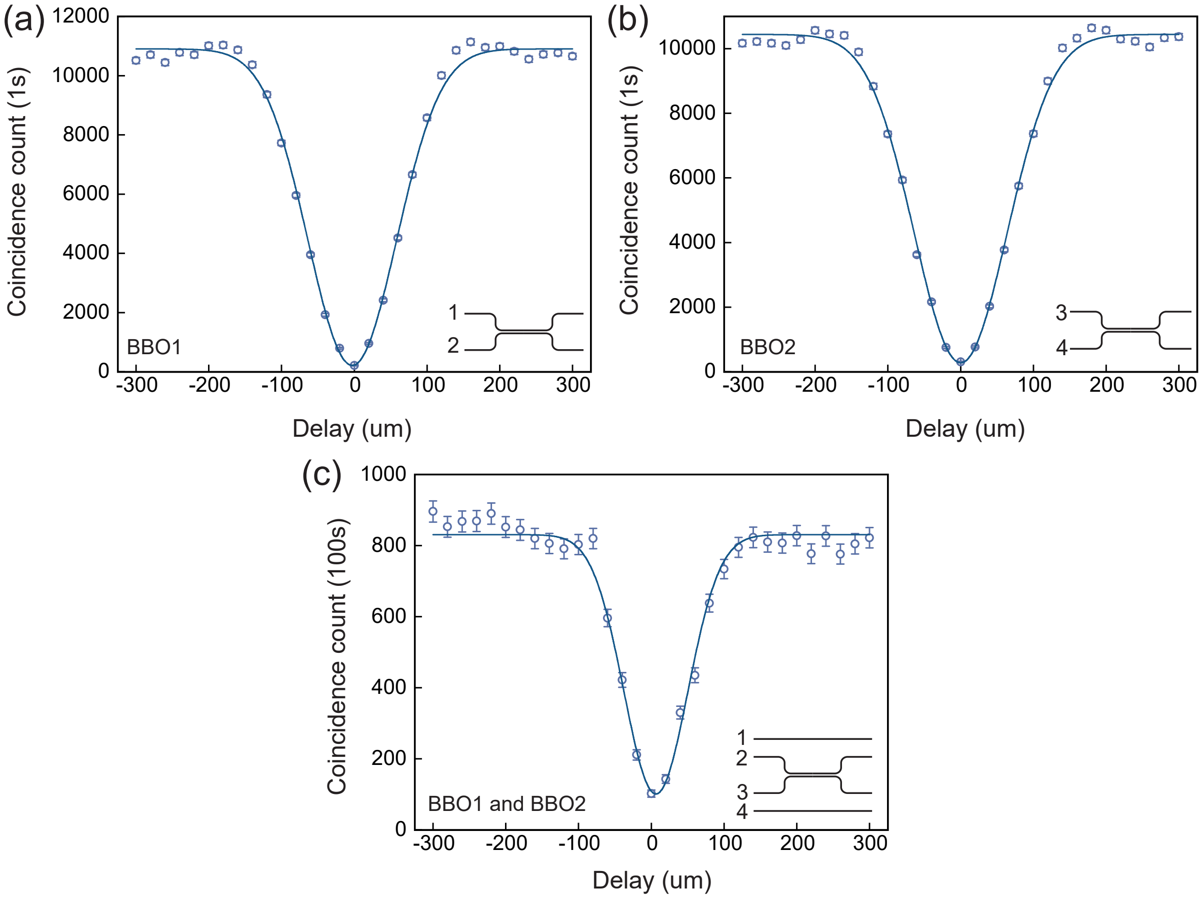

Figure S1: **Interference visibility of the quantum sources (a)**Interference visibility of correlated photons generated by BBO1. (**b**)Interference visibility of correlated photons generated by BBO2. (**c**)Interference visibility of synchronizing two pairs of quantum sources.

BBO1 (collinear phase-matched) is $98.03\% \pm 0.14\%$, and the interference visibilities of BBO2 (beam-like phase-matched) is $97.34\% \pm 0.16\%$. When the laser pump power is 350 mW, the interference visibilities of synchronizing two pairs of quantum sources is $87.83\% \pm 1.28\%$. Compared with the interference visibilities of individual source, the interference visibility decreases after synchronizing two pairs of quantum sources. The decrease is primarily attributed to the deviations in spectra and polarizations among different photons. In order to complete the three-photon experiment within a reasonable execution time, the laser power during the experiment is locked at around 600 mW. At this time, the brightness of the two sources is approximately 100 kHz, and the 4-fold coincidence rate is around 120 Hz.

Moreover, in our experiment, the polarizations of photons are initialized to horizontal polarization. The extinction ratio (H:V) measured after passing through the polarization-maintaining optical fiber exceeds $50 : 1$, while the extinction ratio after passing through the chip waveguide is around $10 : 1$. The reduction in interference visibility and polarization extinction ratio impacts multi-photon correlation measurements, leading to a decline in experimental fidelity.

S.2 Deviation analysis of the waveguide evolution length and single-photon intensity distributions

We divide this section into two parts. The first part is the determination of the chip evolution length. We simulate the intensity distribution at different evolution lengths when the input is site 9. In Fig.S2(a), we observe that site 4 and site 8 are the sites at the edges of the triangular lattice. Fig.S2(b) further illustrates the probability of finding a photon at site 4 and site 8 with different evolution lengths. We can find that the intensity at site 4 peaks within the range of 2.5 mm to 3 mm, indicating full evolution of the single photon within the lattice without bouncing off the edges. Subsequently, we fabricate the samples and select a group (2 mm in the experiment and 2.5 mm in the simulation) exhibiting higher similarity for realizing multi-

photon quantum walks experiments. The deviation in evolution length between experiment and simulation mainly comes from the output bending transformation in the chip structure, leading to additional evolution length. While we use the differential element method in the simulation to compensate for additional evolution length, some deviations persist.

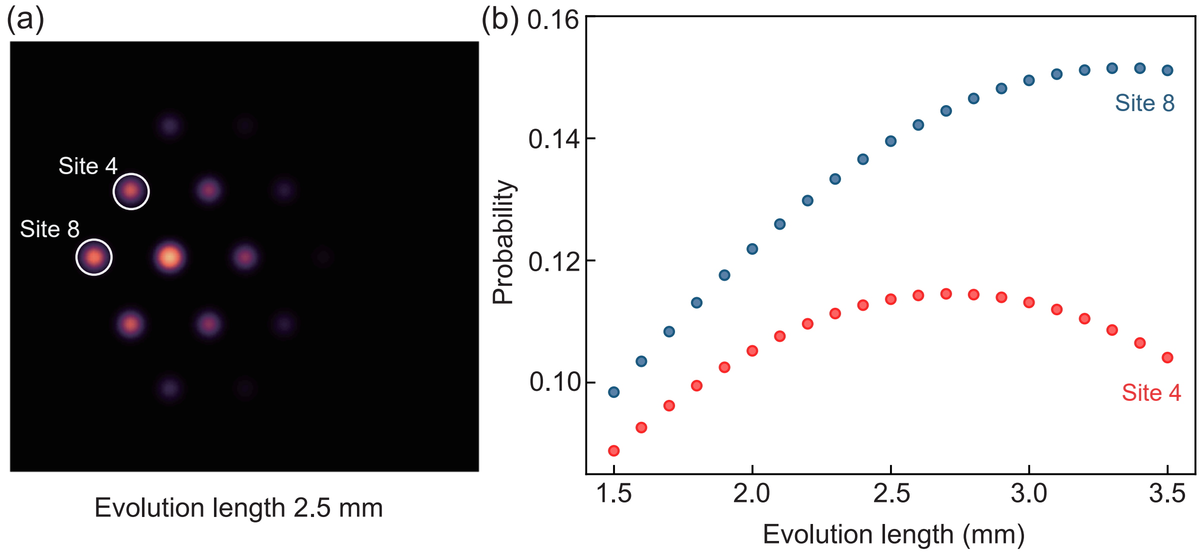

Figure S2: **Intensity distribution with different sites and evolution lengths (a)**Intensity distribution with input site 9 when the evolution length is 2.5 mm. **(b)**The probability of finding a photon at site 4 and site 8 with different evolution lengths.

The second part is the deviation between the experimental and simulated intensity distribution (Fig.2(e) and Fig.2(h) in the main text), which can be attributed to two main reasons. Firstly, we present the intensity distributions obtained through different detection methods in Fig.S3. We further analyze the intensity proportion of the central site 10. Upon comparing Fig.S3(b) and S3(c), the intensity proportion directly detected by the CCD camera has a result of 11.1%, whereas the intensity proportion detected by the external fiber array and avalanche photodiodes (APDs) resulted in 8.2%. This indicates that the connection between the chip and external fiber array introduces additional losses. Secondly, as a result of the connection with a standard external fiber array, the chip evolution region gradually transitions to the output waveg-

uide. This process also adds additional coupling. Upon comparing Fig.S3(a) and S3(c), the light intensity is further coupled outward from the center.

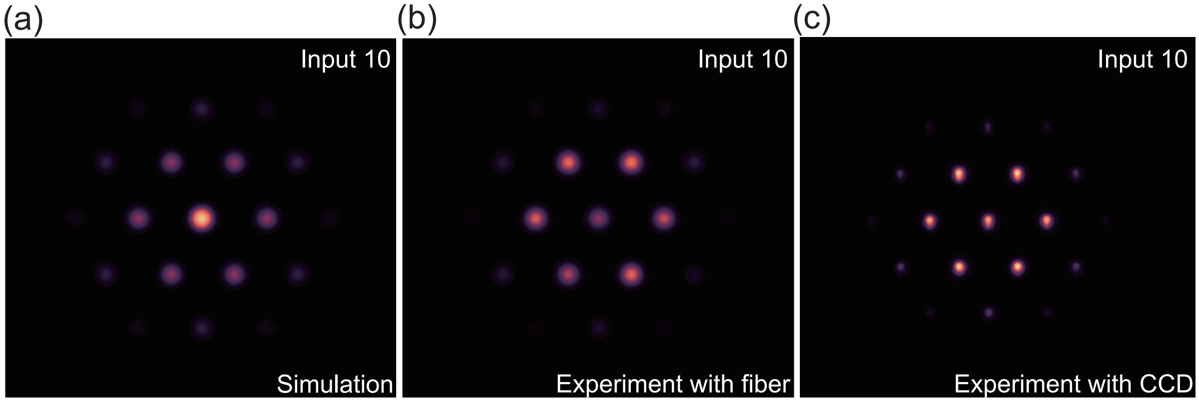

Figure S3: **Intensity distribution with different detection methods (a)**Intensity distribution with simulation. **(b)**Intensity distribution with fiber. **(c)**Intensity distribution with CCD camera.

The deviation of intensity distribution between experimental and simulated results impacts the correlation measurement results of collision-free events and bunching events in Fig.3 of the main text. This inconsistency directly contributes to a decrease in the fidelity of the results. To tackle these problems, firstly, the validation method we use in our manuscript is based on the statistical signatures of the probability distribution. This method reflects the holistic quantum features of correlation measurements rather than focusing on specific values, rendering it resilient to the deviations. Secondly, in order to further improve the fidelity of the experiment, we are working on implementing an imaging-based large-scale correlation measurement (Tpx3), which does not require additional bending transformations and external fiber arrays.

S.3 Measurement and reconstruction of the quantum bunching events

Photon number resolution is very important for multi-photon interference and quantum walks because quantum bunching effects can be observed by directly measuring the results of photon number resolution. The most convenient method is to use photon number-resolving detectors

which have been shown in Fig.S4(a). We use the output (i, j, k) of the chip as an example, the combinations can be divided into three conditions: three photons in three different modes; two photons in one mode and three photons in the same mode. However, photon number-resolving detectors are still the challenging and scarce technology. Another efficient method is to separate the photons into auxiliary modes by using the cascade of fiber beamsplitters. As shown in Fig.S4(b), with this approximate photon number-resolving detection, we need 19 sets of such cascade of fiber beamsplitters and 57 detectors, which results in additional fiber connection losses and cost, thereby reducing the count rate of the experiment and making the experiment more time-consuming and expensive compared with photon number-resolving detectors. To make the experiment completed within a reasonable count rate and execution time, we divide the detection into three steps:

I. For the collision-free combinations, the measurement method is not different from that in Fig.S4(a), where three photons are detected in three different modes i, j, k.

II. For the two-photon bunching combinations, as shown in Fig.S4(c), we add a balanced beamsplitter (1:1) behind one of the outputs i and measure 18 bunching combinations $N_{i,i,k}$ for 15 hours simultaneously. The efficiencies of the two arms of the beamsplitter are γ_{bs1} and γ_{bs2}, therefore, the rescaled coincidence counts $N^{re}_{i,i,k} = \frac{N_{i,i,k}}{\gamma_{bs1}\gamma_{bs2}}$. Then we switch to another output and measure the coincidence counts group by group. Eventually we measure enough coincidence counts (7 groups) in the first layer of sites and normalize the probability distribution in the subspace as Fig.3(b) in the main text shows.

III. For the three-photon bunching combinations, as shown in Fig.S4(d), we add a cascade of fiber beamsplitters (approximate 1:1:1) behind one of the outputs j and measure the bunching combinations $N_{j,j,j}$ for 50 hours. We only measure 23 classical coincidence counts and 65 quantum coincidence counts on site 10. Similarly, the rescaled coincidence counts $N^{re}_{j,j,j} = \frac{N_{j,j,j}}{\gamma_{bs1}\gamma_{bs2}\gamma_{bs3}}$.

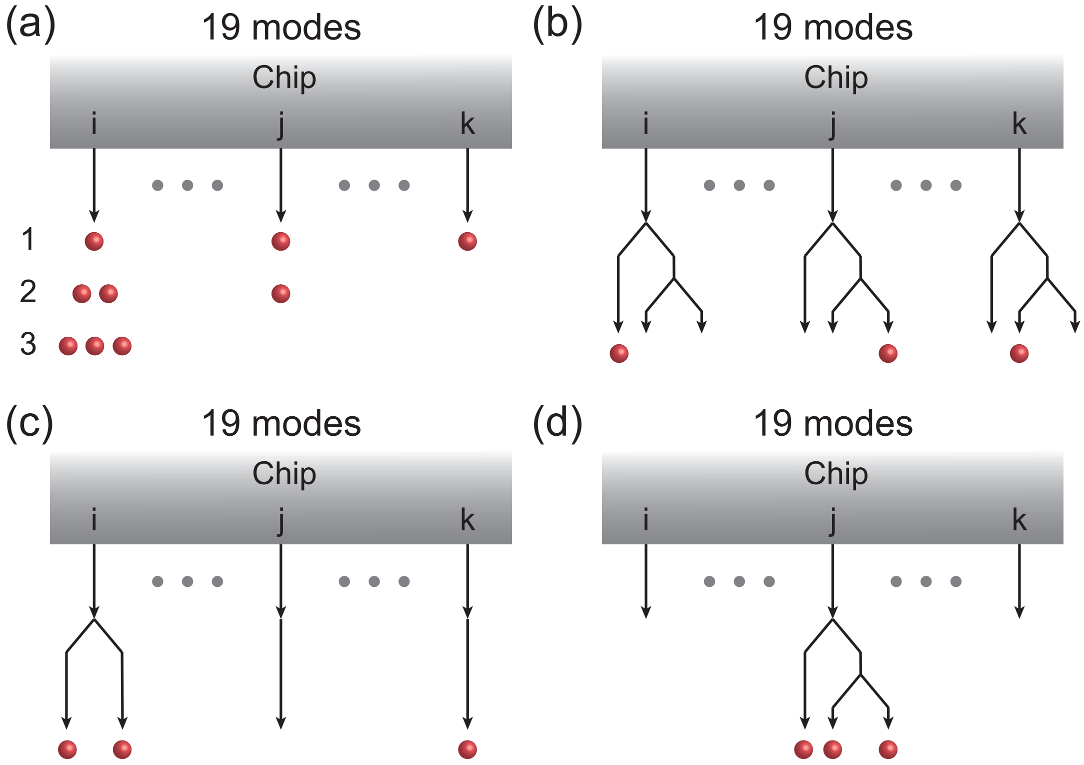

Figure S4: **Schematic diagram of the experimental setup for photon number resolution** **(a)**Measurement method with photon number-resolving detectors. **(b)**Measurement method with the cascade of fiber beamsplitters to separate the output photons in different modes. **(c)-(d)**Measurement method in our experiment with limited fiber beamsplitters and avalanche photodetectors (APDs).

Moreover, the deviation of the experiment mainly comes from the imperfect beamsplitter ratio and the different losses among different modes, which makes the reconstructed counts have a certain offset relative to the theoretical value. Therefore, an efficient multi-photon interference validation method is necessary for all high-dimensional quantum simulation experiments.

Furthermore, for a three-photon experiment with m mode number, the proportions of 2-photon bunching combination is $\frac{6(m-1)}{(m+1)(m+2)}$, and the proportions of 3-photon bunching combination is $\frac{6}{(m+1)(m+2)}$. The proportion of bunching events decrease rapidly as mode number increases. Therefore, as the scale of the experiment increase, the complete measurement of all the bunching combinations is challenging.

S.4 Simulation of the statistical signatures on C-dataset

We perturb the positions of the site to generate the C-dataset. As shown in Fig.S5(a), we use the central site to illustrate the method. The lattice constant $a = 15\ \mu m$ in the evolution region. Considering the actual experimental situation, in order to prevent the light spot of the two adjacent waveguides from overlapping, the distance between the fabricated waveguides is usually not less than $8\ \mu m$, so the max oblique perturbation range of the central site $P = [-(a-8)/2\ \mu m, (a-8)/2\ \mu m]$. We define the degree of perturbation $\eta = P'/P$, where P' is the actual perturbation range. Obviously, when η is larger, the range of perturbation is large and the randomness of the system is large. The perturbation range in the x direction is $P_x = P/2$ and the perturbation range in the y direction is $P_y = \sqrt{3}P/2$. Then, we scan the coupling strength for different waveguide spacings and the results have been shown in Fig.S5(b). For a certain η, we simulate 5000 quantum walks with different Hamiltonians and obtain 5000 sets of C-dataset. Then we can calculate statistical signatures on C-dataset.

We further discuss the simulation results with different interference visibilities. The interference visibilities can be altered by adjusting the ratio of the permanent and determinant[1].

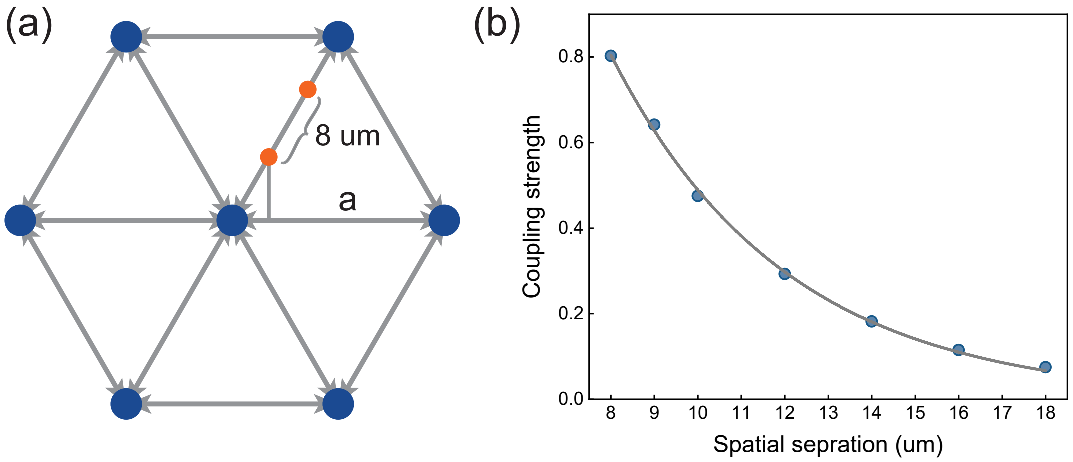

Figure S5: **(a)**Schematic diagram of site perturbation range. **(b)**Coupling strength for different waveguide spacings.

By adjusting the ratio between quantum correlations and classical correlations, we supplement the simulation results with different interference visibilities on the (NM, CV) plane at a perturbation degree of $\eta = 0.5$, as shown in Fig.S6. The blue region represents quantum behavior (100% interference visibilities), the red region represents classical behavior (0% interference visibilities), and the green region represents a combination of 50% quantum behavior and 50% classical behavior. The ratio can also correspond to 100% permanent (blue), 50% permanent and 50% determinant (red), and 75% permanent and 25% determinant (green). It is evident from Fig.S6 that decreasing interference visibilities result in the quantum region converging towards the classical region, thereby complicating the differentiation between quantum and classical correlations. Since the experimental results will be classified into these regions, therefore, the interference visibilities will influence the simulation and experimental results. In the experiment, we can change the interference visibilities by adjusting the delay between two pairs of sources.

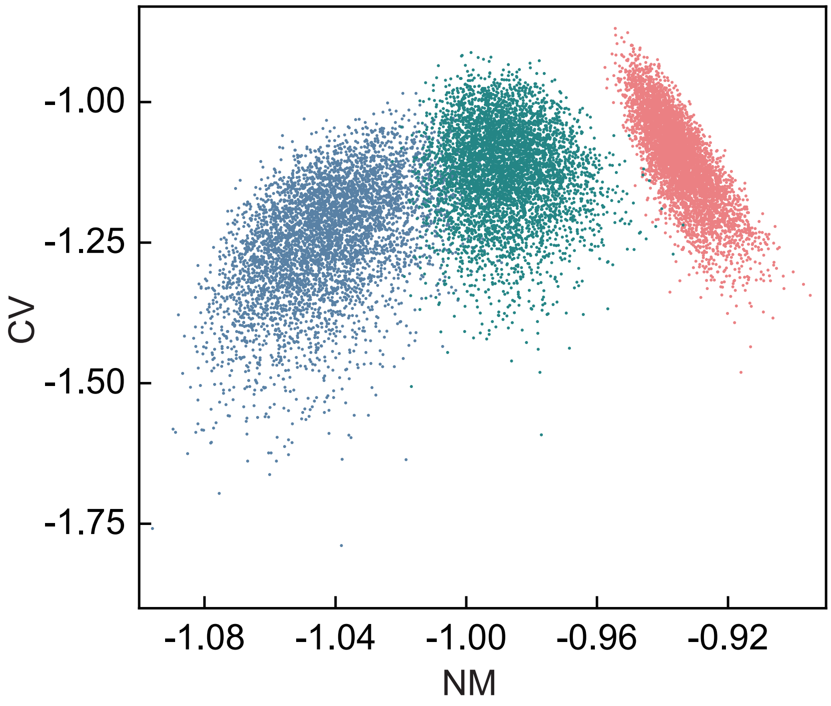

Figure S6: **Simulation results with different interference visibilities**

S.5 Three-photon bunching effect on a single marked site.

We simulate the probability of the combination (10,10,10) with different evolution lengths in Fig.S7, using the inputs identical to those in the main text. We can find that theoretically the probability of quantum correlation is always 6 times than classical correlation. The three-photon bunching effect can enhance the search efficiency on a single marked site. We extend the quantum walks to the many-body level for the first time, potentially unveiling new and unexplored application possibilities that warrant to be further research.

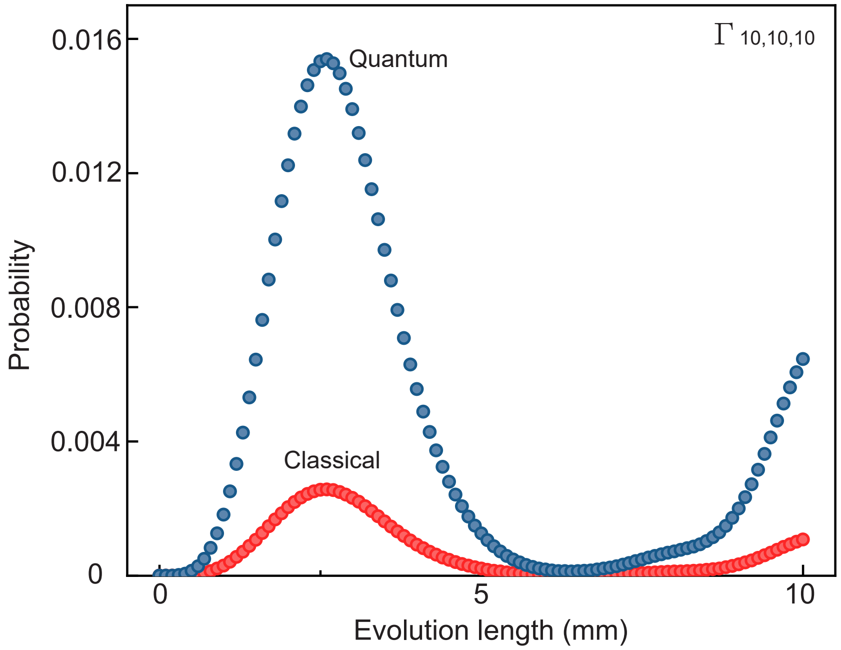

Figure S7: Three-photon bunching effect on a single marked site.

References

[1] M. Tillmann, *et al.*, *Physical Review X* **5**, 041015 (2015).
